# On the way to specificity ‐ Microbiome reflects sponge genetic cluster primarily in highly structured populations

**DOI:** 10.1111/mec.15635

**Published:** 2020-09-30

**Authors:** Cristina Díez‐Vives, Sergi Taboada, Carlos Leiva, Kathrin Busch, Ute Hentschel, Ana Riesgo

**Affiliations:** ^1^ Department of Life Sciences The Natural History Museum London UK; ^2^ Departamento de Ciencias de la Vida EU‐US Marine Biodiversity Group Universidad de Alcalá Alcalá de Henares Spain; ^3^ Departamento de Biología (Zoología) Universidad Autónoma de Madrid Facultad de Ciencias Madrid Spain; ^4^ Department of Genetics, Microbiology and Statistics Faculty of Biology University of Barcelona Barcelona Spain; ^5^ GEOMAR Helmholtz Centre for Ocean Research Kiel Research Unit Marine Symbioses Kiel Germany; ^6^ Department of Biodiversity and Evolutionary Biology Museo Nacional de Ciencias Naturales de Madrid (CSIC) Madrid Spain

**Keywords:** host‐specificity, microbiome, sponges

## Abstract

Most animals, including sponges (Porifera), have species‐specific microbiomes. Which genetic or environmental factors play major roles structuring the microbial community at the intraspecific level in sponges is, however, largely unknown. In this study, we tested whether geographic location or genetic structure of conspecific sponges influences their microbial assembly. For that, we used three sponge species with different rates of gene flow, and collected samples along their entire distribution range (two from the Mediterranean and one from the Southern Ocean) yielding a total of 393 samples. These three sponge species have been previously analysed by microsatellites or single nucleotide polymorphisms, and here we investigate their microbiomes by amplicon sequencing of the microbial 16S rRNA gene. The sponge *Petrosia ficiformis*, with highly isolated populations (low gene flow), showed a stronger influence of the host genetic distance on the microbial composition than the spatial distance. Host‐specificity was therefore detected at the genotypic level, with individuals belonging to the same host genetic cluster harbouring more similar microbiomes than distant ones. On the contrary, the microbiome of *Ircinia fasciculata* and *Dendrilla antarctica* ‐ both with weak population structure (high gene flow) ‐ seemed influenced by location rather than by host genetic distance. Our results suggest that in sponge species with high population structure, the host genetic cluster influence the microbial community more than the geographic location.

## 1 INTRODUCTION

Sponges (phylum Porifera) are early‐divergent metazoans that represent a major part of the marine benthic fauna across the world's oceans, providing fundamental services to the ecosystems where they live (Bell, 2008; Taylor et al., 2007). Despite their relatively simple body plan, sponges are known for hosting complex, dense, diverse, and highly specific microbial communities (Thomas et al., 2016). In some cases, these microbial associates comprise as much as 90% of the sponge volume and can contribute significantly to host metabolism and biochemical repertoire (Taylor et al., 2007; Webster & Thomas, 2016). Each sponge species harbours a specific symbiotic community, resulting from the combination of two proposed acquisition mechanisms: environmental (horizontal) acquisition of microbes, and parental (vertical) transmission (Björk et al., 2019; Sipkema et al., 2015; Taylor et al., 2007). This host species‐specific nature of sponge‐associated microbial communities is now well‐established, with most studies reporting the “host identity” (i.e., host species) to be the single strongest influence on the composition of the associated microbial community (O’Brien et al., 2019; Reveillaud et al., 2014; Thomas et al., 2016). The sponge phylogeny (“host relatedness”), however, is not unequivocally linked with the microbial similarity. Sponge relatedness has been associated with microbial diversity and community composition in some studies (Easson & Thacker, 2014; Schöttner et al., 2013; Souza et al., 2017; Thomas et al., 2016). Contrastingly, other studies reported closely related sponges harbouring different microbial communities (Easson & Thacker, 2014; Schmitt et al., 2012).

Host‐specificity patterns below species level are even less understood, because in almost all cases, research has focused on describing the intraspecific variability of sponges with respect to environmental drivers of variation, and among these studies, results have been inconsistent. In some studies, intraspecific microbial communities differed over spatial and temporal scales, location, nutrient concentration or habitat (Anderson et al., 2010; Burgsdorf et al., 2014; Fiore et al., 2013; Luter et al., 2015; Turque et al., 2010; Weigel & Erwin, 2016). In contrast, other studies reported stable microbial communities over spatial scales, different seasons and depths (Björk et al., 2013; Erwin et al., 2012, 2015; Hentschel et al., 2002; Pita et al., 2013; Reveillaud et al., 2014; Simister et al., 2013; Taylor et al., 2005). Studies including more than one sponge species in different locations also found contrasting findings for the different species included, proposing different strengths in host‐symbiont interactions for different sponge species (Cleary et al., 2013; Lee et al., 2009; Thomas et al., 2016), or an effect of the genetic variability of the sponge host (Taylor et al., 2005). Recently, few studies have revealed an apparent influence of intraspecific host genetics in structuring the microbial communities in single sponge species from the Caribbean (Easson et al., 2020; Griffiths et al., 2019; Marino et al., 2017). However, in the Indo‐Pacific, microbial variation was predominantly related to geography as opposed to host genetic groups (Swierts et al., 2018).

The role of subspecies host genetic divergence in determining the intraspecific variability of the microbial community, therefore, has yet to be largely determined for marine sponges. In other organisms, including animals and plants, the impact of host genetic variation on the microbiome composition has already been reported, including the human gut microbiome (Davenport, 2016; Kolde et al., 2018; Spor et al., 2011), mouse lines (Benson et al., 2010), or plant genotypes (Bouffaud et al., 2014; Qian et al., 2018). In sponges, host genotype variability is only known for some species with the appropriate markers for fine resolution (see review Pérez‐Portela & Riesgo, 2018). In fact, classic analyses with ribosomal or mitochondrial DNA markers are not sensitive enough to detect such the levels of genetic variability. In turn, microsatellites and single nucleotide polymorphisms (SNPs) are powerful genetic markers that provide resolution of population structure and therefore genetic variability at local and global scales for sponges (Leiva et al., 2019; Pérez‐Portela & Riesgo, 2018). Using these techniques to investigate population connectivity, moderate to high gene flow has been detected for sponges (Chaves‐Fonnegra et al., 2015; Giles et al., 2015; Leiva et al., 2019; Riesgo et al., 2016; Taboada et al., 2018), and very rarely, true genetic isolation (low gene flow) of different populations has been reported (Riesgo et al., 2019). Whether this variation in gene flow and genetic divergence has any impact on the composition of the microbiome has never been tested appropriately.

The goal of the present study was to determine the effect of both intraspecific genetic variation and geographic location (over 3,000 km in the Mediterranean Sea, and 740 km in the Southern Ocean) on the microbial community structure and composition of three marine sponge species. We characterized the microbiome compositions of two Mediterranean demosponges (*Ircinia fasciculata* and *Petrosia ficiformis*) and one Antarctic demosponge (*Dendrilla antarctica*), which present different levels of gene flow and *a priori* microbiome acquisition strategies. The connectivity of the sponge hosts had previously been assessed with microsatellite markers for the Mediterranean sponges (Riesgo et al., 2016, 2019) and with SNPs for the Antarctic sponge species (Leiva et al., 2019). We tested whether a possible intraspecific host‐specificity signal is present both across geographic space and host genetic clusters. Our study contributes to refine our understanding of the relationship between host speciation and microbial community composition in sponges, one of the oldest animal phyla on the planet.

## 2 MATERIALS AND METHODS

### 2.1 Sponge sampling

Three different sponge species were used in this study to examine host sponge genetic distance and associated microbial community dissimilarities. Sponge species were collected at different times and locations, and therefore, were not directly compared with each other (Table S1). A total of 168 individuals of *Petrosia ficiformis*, a high microbial abundance (HMA) sponge that displays exclusively horizontal transmission of symbionts (Lepore et al., 1995; Maldonado & Riesgo, 2009), were collected in shallow waters of the Mediterranean Sea along 17 locations during three sampling campaigns in July–August of different years (see details of collection in Riesgo et al., 2019). For the second sponge, *Ircinia fasciculata,* also a HMA sponge that in turn displays vertical transmission (Björk et al., 2019), 166 individuals were collected also in shallow waters of the Mediterranean Sea, along 11 locations in July–August of four different years (see Riesgo et al., 2016 for details). Finally, 62 individuals of *Dendrilla antarctica*, low microbial abundance (LMA) sponge with horizontal transmission (Koutsouveli et al., 2018), were collected from Antarctic shallow waters in seven locations along the Antarctic Peninsula in one single campaign during the 2015–2016 Austral summer (see Leiva et al., 2019). All sponge species were preserved in absolute ethanol that was replaced with fresh ethanol at least three times within 48 hr and stored at −20°C until further processed. DNA was extracted with the DNeasy Blood & Tissue kit (Qiagen, Hilden, Germany) following the manufacturer's instructions with a minor modification concerning overall cell lysis time (that is, incubation was conducted overnight) and the final DNA elution step (performed twice using 50 μl of buffer EB each time).

### 2.2 Genetic distances of the host

Genetic clusters were assigned to individuals based on microsatellite data sets for *P. ficiformis* and *I. faciculata* (Riesgo et al., 2016, 2019), and from single‐nucleotide polymorphisms (SNPs) for *D. antarctica* (Leiva et al., 2019) using a Bayesian clustering approach in STRUCTURE 2.3.4 (Pritchard et al., 2000), that calculates population allele frequencies and then assigns individuals to populations probabilistically (see references Leiva et al., 2019; Riesgo et al., 2016, 2019 for details of the analyses). Then, Euclidean genetic distances among individual sponge samples were calculated with GENODIVE version 2.0b23 (Meirmans & Van Tienderen, 2004) for the microsatellite data sets, and using the *dist* function in R v.2.14 (R Core Team, 2019) for the SNP data set. Finally, population differentiation between pairwise sampling sites and genetic clusters was also estimated with GENODIVE using the *F*_ST_ statistic and an infinite allele model (IAM). Significance of *F*_ST_ values was analysed with 20,000 permutations.

### 2.3 16S rRNA amplicon sequencing

For *P. ficiformis* and *I. fasciculata*, we targeted the V3–V4 hypervariable regions of the 16S rRNA gene, while the V4 hypervariable region was used for *D. antarctica*. The V3V4 region was amplified using a one‐step PCR with the following conditions: 98°C for 30 s, followed by 30 cycles of 98°C for 9 s, 55°C for 1 min, 72°C for 1.5 min, and a final elongation at 72°C for 10 min. We used the primer pair 341F (Muyzer et al., 1993) and 806R (Caporaso et al., 2011) in a dual‐barcoding approach (Kozich et al., 2013). Verification of PCR‐products was accomplished by electrophoresis on an agarose gel. Normalisation and cleaning was done with the SequalPrep Normalization Plate Kit (Invitrogen). Afterwards products were pooled equimolarly and sequenced on a MiSeq platform using v3 chemistry (2 × 300 bp) at the University Kiel, Germany (https://www.ikmb.uni-kiel.de/).

The V4 region of the 16S rRNA gene was amplified using general bacterial primers 515F‐Y (Parada et al., 2016) and 806R (Apprill et al., 2015), with the Illumina adapter overhang sequences in both primers. These primers contain degenerated bases to remove the previous bias against Crenarchaeota/Thaumarchaeota, and the Alphaproteobacterial clade SAR11. We used the PCRBIO HiFi Polymerase (PCR Biosystems Ltd) under the following conditions: 95°C for 3 min, followed by 25 cycles of 95°C for 20 s, 60°C for 20 s and 72°C for 30 s, after which a final elongation step at 72°C for 5 min was performed. DNA amplification was done in duplicates, and PCR products were checked in 1% agarose gel to determine the success of amplification and the relative intensity of bands. PCR products were purified with AgencourtAMPure XP Beads (Beckman Coulter Inc.), and libraries prepared with the Nextera XT DNA Library Preparation Kit (Illumina Inc.). An equimolar pool of DNA was generated by normalizing all samples at 4 nM for sequencing. Next generation, paired‐end sequencing was performed at the Natural History Museum of London (https://www.nhm.ac.uk/) on an Illumina MiSeq device using v3 chemistry (2 × 300 bp).

### 2.4 Read processing, taxonomic assignment and core ASVs

Raw paired reads were imported into Mothur (v.1.41.3), and an adaptation of the MiSeq SOP protocol was followed (Kozich et al., 2013). Briefly, primer sequences were removed and sequence contigs built from overlapping paired reads. The merged amplicon sequence lengths were ca. 458 bp and 298 bp for the V3–V4 and V4 regions, respectively. Sequences with >0 N bases or with >15 homopolymers were discarded. Unique sequences were aligned against the Silva reference data set (release 132), and poorly aligned sequences removed. Unoise3 (Callahan et al., 2016), which is implemented within mothur, was used for denoising (i.e., error correction) of unique aligned sequences, to infer amplicon sequence variants (ASVs), allowing one mismatch per 100 bp (Oksanen et al., 2018). Any singletons remaining at this stage were removed. Reference based chimera checking was conducted using UCHIME with the Silva reference data set and parameter minh = 0.3. ASVs were classified using the Silva database v.132, with a cutoff value of 80. ASVs classified as eukaryotic‐choroplast‐mitochondria or unknown were discarded, this represented less than 0.002% of sequences for the Mediterranean samples and 0.4% in the Antarctica data set. Sequences that remained unclassified further than to Kingdom bacteria or archaea, accounted for 3.1% of Mediterranean samples, and 13% Antarctic samples. Any sample with less than 1,000 sequences was discarded. Community sampling efficiency was examined using rarefaction curves.

Description of the microbial community was done using the total number of ASVs transformed to relative abundances within each individual. Furthermore, for alpha diversity analyses, samples were rarefied to 5,000 sequences for *P. ficiformis* and *I. fasciculata* (discarding 19 and 29 extra samples that did not reach the minimum, respectively), and a minimum of 11,000 sequences for *D. antarctica* (two samples were excluded). The core microbiome was determined on the rarefied data sets using two definitions: ASVs that were present in 100% of the samples at any abundance, and in 80% of samples at any abundance.

### 2.5 Statistical design and analysis

To analyse the influence of genetic and geographic effect on the microbial community, both continuous and discrete variables were included for statistical analyses. Continuous genetic distances among hosts were calculated as Euclidean genetic distances. Samples with 0 Euclidean distance (i.e., clones) were discarded from further analyses. Continuous geographic distances were calculated as kilometres between sampling sites based on the GPS coordinates of each site. Discrete genetic groups were based on the host genetic clusters defined previously (see Leiva et al., 2019; Riesgo et al., 2016, 2019), and discrete geographic groups were designated at sampling collection level (locations).

Correlation between community composition (Bray–Curtis dissimilarity) and both continuous distances (i.e. host Euclidean distance and geographic distances) were tested using Mantel and partial Mantel tests implemented in the R package vegan. The Mantel approach computes Pearson correlation between continuous distances, with significance based on 999 permutations of the distance matrix. Correlations were analysed globally (including all samples), using partial Mantel test controlling for the effect of a third variable, and also on each location separately and each genetic cluster.

Measures of ASV richness, Shannon index, and inverse Simpson's index were calculated using the rarefied samples in R v.3.6.1. These metrics were compared among genetic clusters using analyses of variance (ANOVA). Pairwise comparisons were conducted using TukeyHSD. Beta diversity was calculated using the Bray‐Curtis dissimilarity coefficient. ASVs were filtered by a relative abundance >0.01% in at least 5% of samples, leaving 1,557, 2,032 and 1,059 ASVs for *P. ficiformis*, *I. fasciculata* and *D. antarctica* respectively. The relative abundances were then log2 transformed prior to calculation of Bray‐Curtis dissimilarities. These dissimilarity matrices were visualised using Principal coordinates analysis (PCoA) using “*cmdscale*” in *vegan* v. 2.5–6 (De Cáceres & Legendre, 2009). We compared distances among genetic clusters and locations (discrete factors) by permutational multivariate analyses of variance (PERMANOVA) using “*adonis*” in *vegan* and a type II of sums of squares for partitioning terms in unbalanced designs. When samples were collected in different years, this factor was added into the analysis. For *P. ficiformis*, beta diversity analyses were re‐run after transforming the data to presence absence (and using Jaccard distances), and at genus level (ASVs abundances belonging to the same genera were aggregated), to see the effect on alternative data sets.

Beta diversity analyses were initially performed on the entire data sets, however, since genetic, geographic and year effect may be confounding, we repeated analyses for each independent location, genetic cluster and year of sampling. In this case, locations with insufficient host genetic variation were excluded, meaning that only locations with more than one identified genetic cluster and with at least three replicates (for *P. ficiformis* and *I. fasciculata*) were considered. In *D. antarctica* we reduced the number to two replicates due to the lower number of samples in the data set. This resulted in the informative locations BLA, LIG and NAP for *P. ficiformis*; CALA, CRO, NAP, TOSS, CAB and ESC for *I. fasciculata*; and CIE, KG, ADE and HM for *D. antarctica*. For the analysis of each host genetic cluster separately, we included only genetic clusters present in more than one location with 2 or 3 replicates, similarly as before. These were Pf3, Pf4, Pf5, Pf6, Pf8 for *P. ficiformis*; If1, If2, If4, If5 for *I. fasciculata*; and Da1, Da2, Da3, Da4 for *D. antarctica*. Moreover, to disentangle genetic and geographic effects, these selected locations and genetic clusters were also analysed together.

### 2.6 Indicator species in *Petrosia ficiformis*

Indicator species analysis (identification of species associated with or indicative of groups of samples) was conducted using the R package *indicspecies* (Dufrene & Legendre, 1997) to identify microbial taxa characteristic of each genetic cluster in *P. ficiformis*. This analysis assesses the strength of the relationship between ASVs abundance and different host genetic clusters by comparing ASVs abundance in microbiotas of one genetic cluster to their abundance in the others. Enrichment values were calculated for each indicator as a log‐transformed ratio of each two groups. The Indicator Value index (Dufrene & Legendre, 1997) is the product of two components: (a) the “the specificity or positive predictive value” as the probability that the surveyed ASVs only belongs to the target genetic cluster; and (b) the probability of finding the ASVs in all samples belonging to the genetic cluster, called “the fidelity or sensitivity” component. The statistical significance of this relationship is tested using a permutation test. Multiple pairwise comparisons were corrected based on the Benjamini–Yekutieli false discovery rate control using “p.adjust” function of the *stats* library package in R.

## 3 RESULTS

### 3.1 Genetic distances and genetic clusters of sponge hosts

**FIGURE 1:**
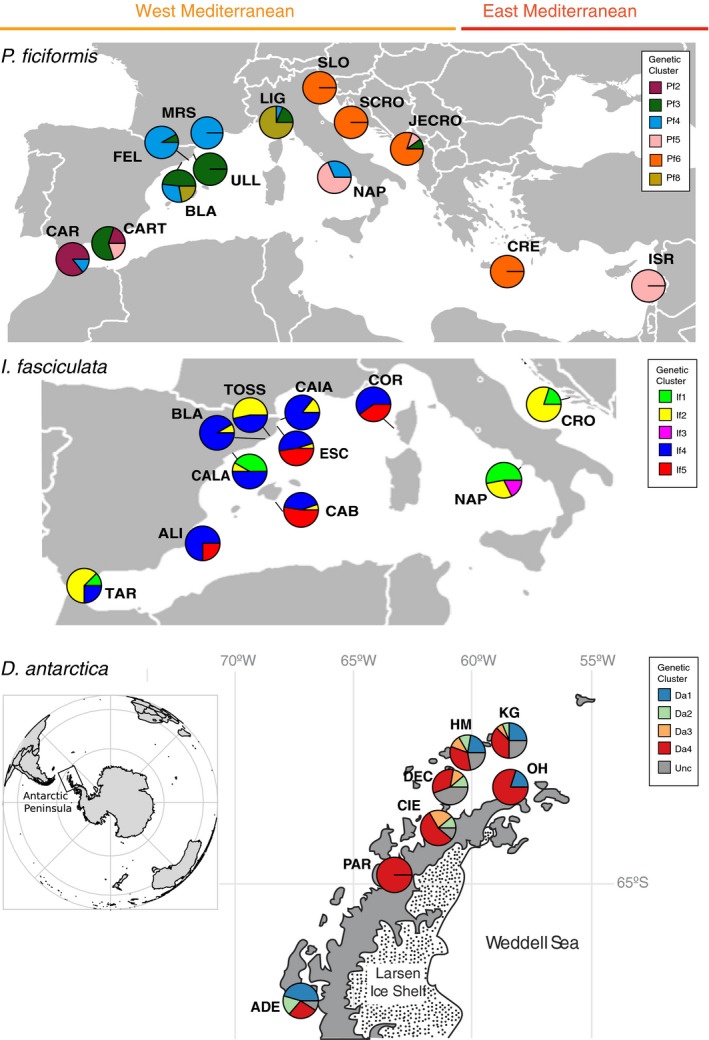
Distribution map of sampling sites over the Mediterranean Sea for *P. ficiformis* and *I. fasciculata* and along the Antarctic Peninsula for *D. antarctica*. The colours of the genetic clusters follow those seen in the original papers (see main text). Location names correspond to: CAR, Carboneras; CART, Cartagena; BLA, Blanes; FEL, Sant Feliu; ULL, Ullastres; MRS, Marseille; LIG, Liguria; NAP, Naples; SLO, Slovenia; SCRO, South Croatia; JECRO, Jelsa Croatia; CRE, Creta; ISR, Israel; TAR, Tarifa; ALI, Alicante; CALA, Calafat; CAB, Cabrera; ESC, Escala; TOSS, Tossa; CAIA, Caials; COR, Corsica; and CRO, Croatia in the Mediterranean Sea; and ADE, Adelaide Island; PAR, Paradise Bay; CIE, Cierva Cove; DEC, Deception Island; HM, Half Moon Island; KG, King George Island; and OH, O'Higgins Bay in the Antarctic Peninsula

**FIGURE 2:**
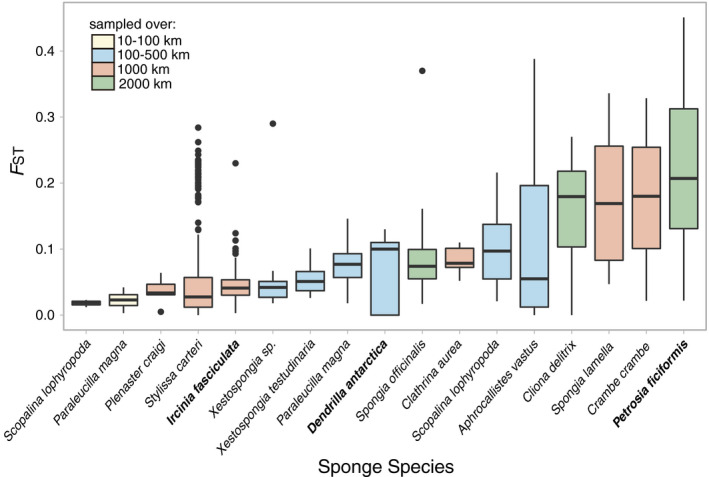
Fixation index values (*F*_ST_) for 17 sponge species taken from the literature. The three sponge species studied here are highlighted in bold. The legend shows a colour coding indicating the geographical span of the sampling used for each species

*Petrosia ficiformis* and *Ircinia fasciculata* were collected across the Mediterranean Sea, including 168 and 163 samples, respectively. *Dendrilla antarctic*a included 62 samples from the Southern Ocean (Figure 1, Table S1). The data set of *P. ficiformis* was grouped into seven genetic clusters (Pf2–Pf6 and Pf8 as reported in Riesgo et al., 2019), five genetic clusters (If1–If5) for *I. fasciculata* (Riesgo et al., 2016), and five genetic clusters (Da1–Da4 plus an unclustered group, i.e., individuals with multiple genetic clusters assigned in which none was dominant over the others) for *D. antarctica* (Leiva et al., 2019). Euclidean genetic distances between individuals for each sponge species can be found in Tables S2–S4. We identified one clone in *P. ficiformis* and three clones in *I. fasciculata* which were discarded from the following analyses, as well as the unclassified group of *D. antarctica*. Fixation indices (*F*_ST_) between genetic clusters were the largest in *P. ficiformis* ranging from 0.048 to 0.276, followed by *D. antarctica* (from 0 to 0.156) and *I. fasciculata* (from 0 to 0.078; Table S5). These *F*_ST_ values can be considered as low, moderate and high gene flow, respectively (Figure 2), when compared to other sponges (Pérez‐Portela & Riesgo, 2018).

**FIGURE 3:**
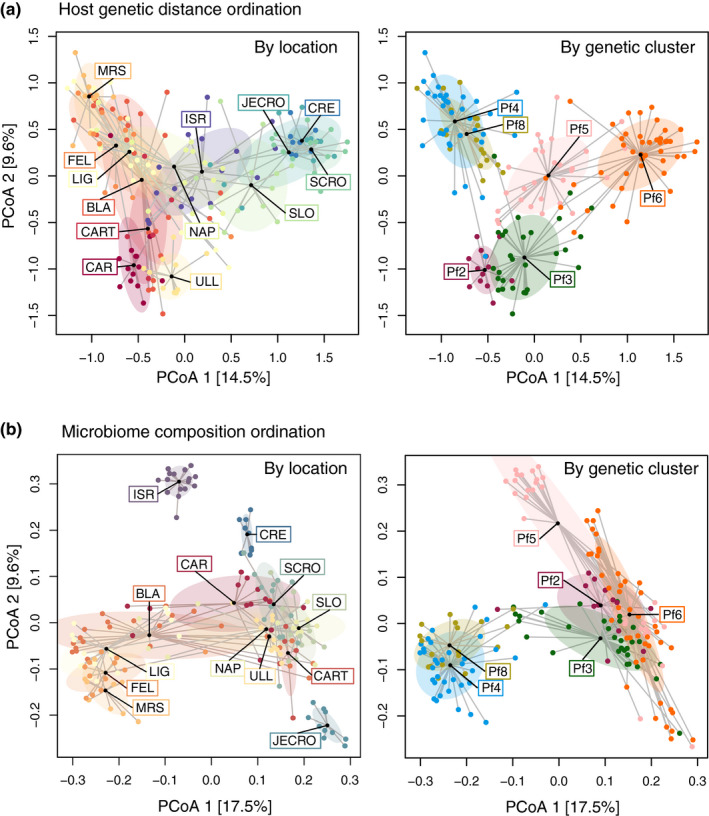
Ordination plots for *Petrosia ficiformis* showing (a) clustering of host genetic distances of all samples coloured by the corresponding location (left side) and by the assigned genetic cluster (right side), and (b) clustering of microbiome dissimilarities of all samples, coloured as in (a). Centroids are marked with their respective factor label and groups are circled with 0.7 data coverage for data ellipses

In *P. ficiformis*, the PCA on the Euclidean distances showed that the first and second principle coordinates explained 24% of the total variation among the samples (Figure 3a). This ordination revealed a clearer clustering of sponge individuals by genetic group rather than by location (i.e., less overlapping of groups). In addition, and in concordance with having the lowest *F*_ST_ values for *P. ficiformis* (i.e., 0.048), genetic groups Pf4 and Pf8 were found to be more closely related than with the rest, presenting overlapping clustering. The PCA in *D. antarctica* explained 26% of the total variation (Figure S1a), showing separation by genetic cluster but not by location, which suggests a stronger host genetic effect over spatial effects. In the case of *I. fasciculata*, the PCA explained 11.8% of the total variation among the samples (Figure S1b). This ordination did not show clear clustering neither by genetic cluster nor by location.

### 3.2 Microbial composition

A total of 107,913, 66,524, and 18,165 unique ASVs were found among all samples of *P. ficiformis*, *I. fasciculata*, and *D. antarctica*, respectively. Abundance values ranged from 1,057 to 115,327 sequences per sample (Table S6). Rarefaction curves showed good representation of amplicon sequences present in *D. antarctica*, but a number of samples did not approach asymptotes for the two other species, suggesting more species would be observed with greater sequencing effort (Figure S2a).

In *P. ficiformis*, the observed ASVs were assigned to 35 phyla and 354 genera. The most abundant phyla were the Chloroflexi with 28.1% mra (mean relative abundance), Proteobacteria (25.4% mra) and Acidobacteria (10.6% mra; Figure S2b). Dominant classes and orders can be found in Table S7. Interestingly, the two most abundant individual ASVs belonged to the phyla Nitrospira and Dadabacteria (2.9% and 2.5% mra, respectively), which were not among the dominant phyla. Looking at the core microbiota, only two ASVs were shared across all samples, but a total of 55 ASVs were shared in 80% of all individuals, and these represented from 18.9% to 47% of the total abundance of the microbiome (75% of samples had at least 30.5% mra). The core included nine of the most abundant phyla (Table S7), with Acidobacteria, Chloroflexi and Proteobacteria including 67%, 34% and 28% of its original abundance among the core ASVs respectively. We also checked the relative abundances of ASVs that were unique to specific collection sites (site‐specific ASVs), and these represented relatively low percentages, i.e., 3.04 ± 1.16% mra, with maximum values found in ISR (Table S8).

For *I. fasciculata*, the observed ASVs were assigned to 34 phyla, and 613 genera. The most abundant phyla were the Proteobacteria (36.3% mra), Cyanobacteria (19.4% mra) and Chloroflexi (18.7% mra; Figure S2b, Table S9). The two most abundant ASVs belonged to Cyanobacteria and Dadabacteria (16.1 and 4.4% mra, respectively). Regarding the core microbiota, one ASV was present in all samples, and 37 ASVs were present in 80% of all samples, and these represented from 7.8% to 71.8% of the total abundance of the microbiome (75% of samples had at least 41.8% mra). The core included 11 of the most abundant phyla (Table S9). The phylum Cyanobacteria represented 19.4% mra, and its most abundant ASV (17.4% mra) was among the core bacteria. The phylum Chloroflexi, however, with a similar mean relative abundance (18.7%) was not so faithfully shared among all individuals, with a core of 4 ASVs including only 3.2% mra. Proteobacterial ASVs were conserved around a 44.5% of their original abundances in the core. Site‐specific ASVs represented 4 ± 3.78% mra (Table S8), which included higher values and larger variability than *P. ficiformis* samples.

ASVs in *D. antarctica* were assigned to 50 phyla, and 913 genera. The most abundant phyla were the Proteobacteria (64.5% mra), Bacteroidetes (16.0% mra) and Verrucomicrobia (3.2% mra; Figure S2b). The remaining phyla had less than 1.5% mra (Table S10). The core microbiota was constituted by four ASVs present in all 62 samples, and 41 ASVs in more than 80% of the samples. The percentage these represented varied from 19.2% to 74.7% of the microbial community abundance (75% of samples had at least 50.7%). The most abundant core ASVs belonged to Proteobacteria representing 35.5% mra, which was 55.2% of the total Proteobacteria abundance in the sponge. *Dendrilla antarctica* site‐specific ASVs represented the lowest values of the three species with 0.79 ± 0.78% mra (Table S8).

Because the sponges *P. ficiformis* and *I. fasciculata* were amplified with different primers than *D. antarctica*, a direct comparison between the ASVs data sets was not possible. Therefore, we used the taxonomic annotation (at the genus level) to look at general differences between the species knowing the limitations that this approach represented. *P. ficiformis* and *I. fasciculata* shared most of the genera, while *D. antarctica* hosted different ones (Figure S2c), which was also reflected in the distance among the samples in a PCoA plot (Figure S2d). However, the specific microbial ASVs within these genera were different among *P. ficiformis* and *I. fasciculata* (Figure S2e), which is in agreement with their microbiomes being species‐specific.

### 3.3 Correlation of host genetic distance and spatial distance with microbial dissimilarity

**FIGURE 4:**
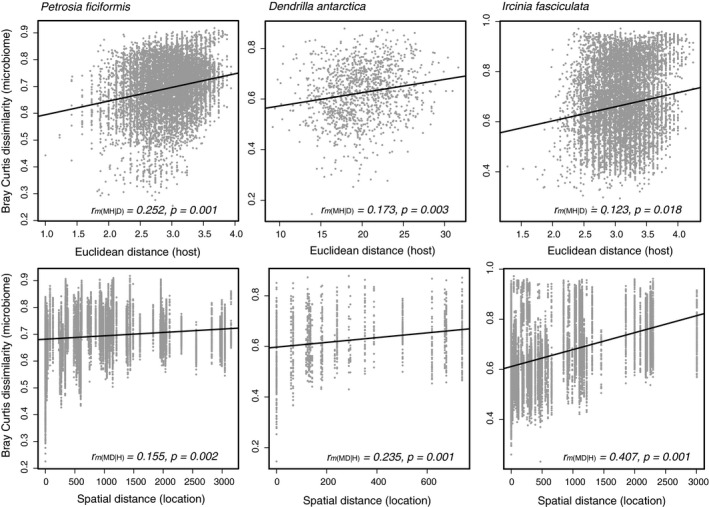
Correlation plots of microbiome dissimilarity (Bray Curtis) versus host genetic distance (Euclidean distance) corrected by the spatial distance on the top row (MH|D); and microbiome dissimilarity (Bray Curtis) versus spatial distance (kilometres between locations) corrected by host genetic distances on the bottom row (MD|H), for the three sponge species. Mantel test statistic (Pearson's *r*) and *p*‐values (*p*) are shown inside each plot

When comparing full data sets of host genetic distances and microbial dissimilarity, Pearson's *r* values from the partial Mantel tests ranged from 0.123 to 0.252 (Table S11), indicating moderate correlation signals, and large variability of the samples (Figure 4). Previous ecological simulations indicated that with moderate‐strong signals, Pearson's *r* were ca. 0.2 on average (Mazel et al., 2018). In turn, Pearson's *r* values for the comparison between geographical distances and microbial Bray‐Curtis dissimilarity values ranged from 0.155 to 0.407 (Figure 4, Table S11). Comparing the effects of both host genetic distance and spatial distance on the microbial community, *P. ficiformis* showed a stronger correlation with the host genetic distance, while in *D. antarctica* and *I. fasciculata* the correlation was stronger with the geographical distances between locations (Figure 4, Table S11).

To remove the bias of the geographical location and year, we analysed the effect of phylosymbiosis in each site independently and each sampling year. In *P. ficiformis*, Pearson's *r* values ranged from −0.33 to 0.64. Four out of the 14 locations for *P. ficiformis* showed strong correlations (*r_m_*_(MH) _> .30, with *p* < .01) between the microbial community Bray‐Curtis dissimilarity and host genetic distance, these were BLA_2013, LIG_2008 and NAP_2013, and marginally BLA_2006 (*p* = .03; Figure S3a and Table S11). The remaining 10 locations, however, included a dominant single genetic cluster (meaning that if more than one genetic clusters were present, the other ones had less than three replicates), and, in those cases, the genetic variation was probably insufficient to detect any correlation. In *D. antarctica* Pearson's *r* values ranged from −0.38 to 0.99. One out of the seven locations for *D. antarctica* (ADE) had high significant correlation (*r_m_*_(MH) _> .40, *p* < .01) between the microbial dissimilarity and the host genetic distance. Three other locations, CIE, KG and HM, presented multiple genetic clusters but did not show significant positive correlation (Figure S3a and Table S11). For *I. fasciculata,* Pearson's *r* values ranged from −0.46 to 0.20, and two out of fifteen locations (i.e. CALA and NAP) had high correlations (*r_m_*_(MH) _> .50, *p* = .001; Figure S3a and Table S11). Similarly as before, another two locations, CRO_2013 and TOSS_2010, presented multiple genetic clusters but no correlation was detected. The rest of locations included a dominant single genetic cluster, and therefore low genetic variance.

Similar results were observed when considering the host genetic clusters independently and testing the effect of geographical distance over microbiome dissimilarity (Figure S3b and Table S11). All the genetic clusters including multiple locations had medium to strong correlation for *P. ficiformis*, while for *D. antarctica* only Da1 showed correlation (*r_m_*_(MD)_ = .30, *p* = .002), and for *I. fasciculata* two of the three genetic clusters including multiple locations showed high correlation (*r* > .40, *p* < .05; Figure S3b and Table S11).

### 3.4 Analysis of variance of microbial communities by genetic cluster and locations

#### 3.4.1 Alpha diversity

The Shannon index was used to unravel whether the genetic cluster or the locations exhibited different diversity patterns. The alpha diversity ranged between 4.42–6.22, 2.14–5.66, and 2.16–4.98 for *P. ficiformis*, *I. fasciculata* and *D. antarctica*, respectively (Table S6 and Figure S4). Diversity was significantly different among genetic clusters of *P. ficiformis* (ANOVA, *F*_12,136_ = 9.73, *p* < .001); and *I. fasciculata* (ANOVA, *F*_10,123_ = 3.28, *p* < .001; Table S12), due to pairwise differences between Pf4 and Pf5 in the former, and between If4–If3 and If4–If5 in the later. Furthermore, the alpha diversity across locations was also significantly different for these two species (ANOVA, *F*_6,141_ = 4.03, and *F*_4,129_ = 8.88, *p* < .001, Table S12), which was associated with JECRO having lower diversity values compared to all other locations in *P. ficiformis*, and between CALA and NAP in *I. fasciculata*. *Dendrilla antarctica* showed no significant differences associated with either genetic cluster or location (ANOVA, *p* > .01).

#### 3.4.2 Beta diversity

An ordination of the microbial community composition by location and genetic cluster is shown in Figure 3b and Figure S1c–d. For the Mediterranean sponges, in general, samples from the Eastern Mediterranean waters harboured different communities than the Western areas. Examples are ISR, CRE and JECRO for *P. ficiformis* that appeared separated from the rest of the samples (Figure 3b), and CRO and NAP for *I. fasciculata* that clustered away from the rest of samples (Figure S1d). In *D. antarctica*, samples from KG, ADE and DEC harboured more different communities than the other four locations (Figure S1c). The microbiomes of the *P. ficiformis* sponges showed separation of samples by genetic cluster (Figure 3b), with a noteworthy overlap between Pf4 and Pf8 samples. This pattern was also observed in the ordination plot of the host Euclidean distances by genetic cluster (Figure 3a), which is here mirrored by their microbial community. Ordination coloured by location showed a large overlap of samples. For *D. antarctica* and *I. fasciculata* in general groups were not clearly separated neither by location nor genetic cluster (Figure S1c,d). Differences in the number of observed genetic clusters in each location, and possible confounding factors occurring among the variables, prevented the statistical analyses using all samples.

**FIGURE 5:**
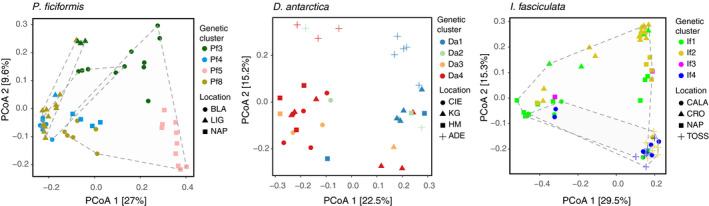
Ordination plots for selected (i.e. informative) locations and genetic clusters in the three sponge species. Samples are given symbols by the corresponding location, colours display the assigned genetic cluster, and dotted lines circle samples from different years

Therefore, in order to perform a factorial analysis of the effect of the host genetic cluster and the spatial distance on the microbial composition, we subset the data set to contain only samples that allowed meaningful comparisons (see methods). This means that for *P. ficiformis* we used only independent locations containing more than one genetic cluster with at least three replicates (namely BLA, LIG, and NAP), and there was a clear grouping of samples associated with their host genetic cluster (PERMANOVA test confirmed significant clustering; Figure S5a and Table S13a). Moreover, within individual genetic clusters, samples diverged according to their sampling location (Figure S5b and Table S13a). To unravel which factor contributed more to the variance, we constructed a data set including BLA, LIG and NAP, and the genetic clusters Pf3, Pf4, Pf5, and Pf8. Generally, there was a clear clustering of genetic groups of *P. ficiformis* regardless of location, with the expected overlap of genetic clusters Pf4 and Pf8 (Figure 5). Both, location and genetic cluster, influenced the microbial communities (Table S13b), but the host genetic cluster had a stronger effect, explaining 19.9% of the variability on the microbiome composition, while location explained 3.4% and year 3.9%, and the interaction of these factors 4.8%. In pairwise comparisons, all genetic clusters were different to each other (*p* = .001) except for the pair Pf4–Pf8 (*p* = .011; Table S13). To further test whether differences in the microbial community were driven by specific assemblages of ASVs or by differences in relative abundances of shared ASVs, we repeated the analysis using Jaccard distances on the presence/absence of ASVs (Figure S6a). We also tested the differences at genus level (by aggregating the abundance of ASVs belonging to same genus (Figure S6b). These two analyses showed that genetic cluster was still the dominant factor compared to location or year, but the effect was less strong than when accounting for relative abundances at ASVs level (i.e., 11.9% and 11.8% for the presence/absence and genus level analyses, respectively; Table S13b). Moreover, in pairwise comparisons using genus level, not only the pair Pf4–Pf8 was not significantly different, but also the genetic cluster Pf3 was not different to either Pf4 or Pf8 (Table S13c).

Four locations could be tested independently for *D. antarctica* (CIE, KG, HM and ADE, Figure S5a). Microbial communities were not different between the different genetic clusters for any location (*p* > .01, Table S13a). Within individual genetic clusters, however, microbial communities from both Da1 and Da4 were significantly different among locations (Figure S5b, Table S13a). Combining CIE, KG, HM and ADE, ANOVA results showed that location had a stronger effect explaining 25.4% of variability while genetic cluster explained 13.2%, and the interaction of these factors 15.3% (Figure S1, Table S12b), but pairwise comparisons showed that only Da1 versus Da3 and Da4, and Da2 versus Da4 presented different compositions (Table S13c).

In *I. fasciculata*, the selected data set included six locations (CALA, CRO, NAP, TOSS, CAB and ESC), and genetic clusters If1 to If4 (Figure S5a). Samples were not different by genetic clusters (*p* > .01, Table S13a), except for CAB and ESC (*p* = .001). However, in these last two locations, samples from either genetic cluster were collected in different years (Table S1) preventing the disclosure of the main factor. Among individual genetic clusters, If4 and If5 showed grouping of microbial communities by location (*p* = .001, Table S13a). Combining the locations (CALA, CRO, NAP, and TOSS but excluding CAB and ESC; Figure 5), PERMANOVA indicated that microbial composition was not significantly different by genetic cluster (*p* > .01), but it was by location (*p* = .001), which explained 10.7% of variability.

### 3.5 Specificity of the microbiome within genetic clusters in *Petrosia ficiformis*

Since *P. ficiformis* was the sponge species with the strongest specificity between microbiome and host genetic cluster, our goal was to identify taxa that were more likely to be found in a given genetic cluster than in others. The R package *indcspecies* identified 725 indicator ASVs among single specific host genetic clusters of *P. ficiformis*, and 373 ASVs whose patterns of abundance were associated to two genetic clusters (Table S14). The genetic cluster Pf2 included 328 indicator ASVs, Pf5 had 188, and Pf4 + Pf8 had 112. Indicator ASVs belonged mostly to the phyla Chloroflexi (364) and Proteobacteria (308), which are in fact the most abundant phyla among *P. ficiformis* samples, and represented 2.7% and 3.6% mra, respectively.

**FIGURE 6:**
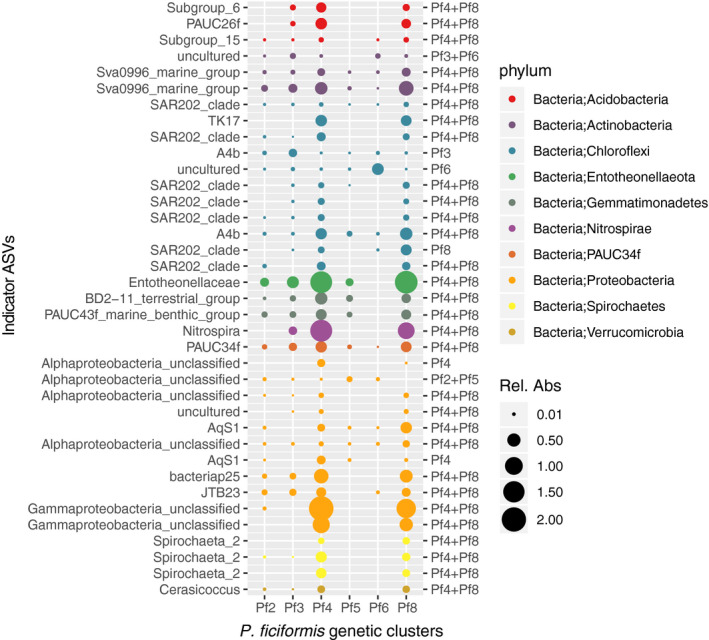
Indicator ASVs for *P. ficiformis* genetic clusters. The figure shows the relative abundance of individual ASVs in each genetic cluster. Labels on the left show the taxonomic classification of each ASV at the genus level, and on the right display what genetic clusters the ASVs are specific to. The colour of the bubbles represents the phylum level for clarity

To further investigate these specificity patterns, we restricted our results to ASVs with FDR < 0.001, specificity of 70% and fidelity of 70%, resulting in 37 indicator ASVs (Figure 6). Interestingly, almost all the indicator ASVs from Pf5 and Pf2 were dropped in this filtering, due to low sensitivity (i.e., not all individuals belonging to the same genetic cluster included them). Within these 37 indicator ASVs, the majority was associated to Pf4 + Pf8 (30 ASVs) plus a few ones in other genetic clusters, and they primarily belonged to phylum Chloroflexi (11 indicator ASVs), and to the phylum Proteobacteria (11 indicator ASVs; Table S14). Among Chloroflexi, ASVs classified as genus SAR202 clade were common in Pf4 + Pf8. It should be noted that these genera, present among the indicator species, also had representatives among the core microbiome, except for genera Spirochaeta 2 and Cerasicoccus (phylum Verrucomicrobia), however, the core ASVs represented a larger relative abundance in the sponge than the indicator ASVs of these genera.

## 4 DISCUSSION

**FIGURE 7:**
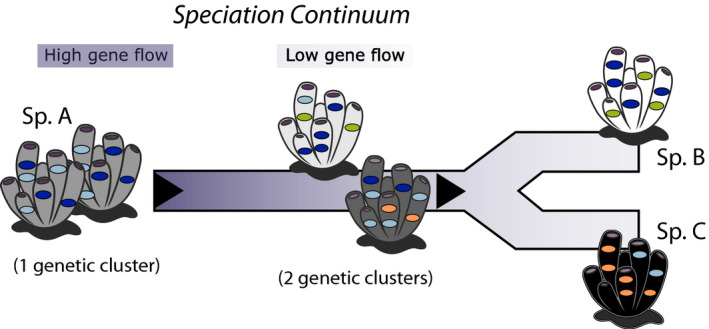
Speciation continuum for the evolution of microbial communities within a sponge species. Sponge individuals are coloured from grey to black or white representing different species or genetic clusters. Microbial symbionts are illustrated as spheres with contrasting colours for different ASVs. Sponge sp. A includes a specific community that is noticeably different in the intermediate genetic clusters (before differentiation in two species) and more distinct as they become fully separated as sponge sp. B and sponge sp. C

Host genetics play a fundamental role in shaping the microbiome of many organisms (Amato et al., 2019; Berg et al., 2016; Brooks et al., 2016; Moran & Sloan, 2015; Qian et al., 2018), but the environment also modulates the composition of microbiomes across different geographical locations (Lurgi et al., 2019; Thompson et al., 2017). To what extent host genetics affects the microbial composition, and what is the interaction between host genetics and environmental factors, are crucial aspects to understand the evolution of microbiomes in eukaryotic organisms. In sponges, host species‐specificity appears to be the strongest factor influencing symbiotic microbial communities, with conspecific sponge species (or phylogenetically related species) from distinct locations sharing a microbial community different from cohabiting species (Thomas et al., 2016). The evolutionary history of a species, however, is not a stepwise or binary process but rather follows a gradual transition (illustrated in Figure 7), known as the “speciation continuum” (Galtier, 2019), which is profoundly shaped by the gene flow within the species (Shaw & Mullen, 2014). Here, we aimed to test whether this continuum may be also detectable in the microbial composition within a given species (i.e., before reproductive isolation), with microbiomes becoming specific prior to speciation splits. Genetic differentiation in sponges is considerably driven by the dispersal capabilities of the sponge species, including both gamete and larvae dispersal potential (Pérez‐Portela & Riesgo, 2018). In this sense, sponge connectivity patterns might have a remarkable effect not only in the survival and adaptation of the sponges themselves, but also in shaping the specificity of the microbiome by influencing their acquisition, recognition, and maintenance.

### 4.1 Effect of host genetics versus geographic location on the sponge microbiome

*Petrosia ficiformis* is an exemplary sponge for investigating the host mechanisms involved in the recognition and maintenance of large consortia of symbiotic communities, given its lack of vertical transmission and the low gene flow between individuals across their distribution range (*F*_ST_ values up to 0.276), which can be taken as a measure of high population differentiation or early speciation (Hey & Pinho, 2012). Moreover, previous studies on *P. ficiformis* concluded that oocytes, sperm and larvae are completely devoid of microbes in this species (Lepore et al., 1995; Maldonado & Riesgo, 2009). This oviparous sponge is known to produce a nonswimming crawling larva, with very limited dispersal potential, accounting for the scarce gene flow detected between their populations (Riesgo et al., 2019). *Petrosia ficiformis* presented a strong pattern of phylosymbiosis, both in global correlations and in the individual locations with sufficient genetic variability, that indicated a more similar microbial community composition for closely related host genetic clusters (populations) than distant ones across the distribution area of the sponge. It is remarkable that mechanisms to acquire and maintain variable specific host‐symbiont associations are displayed within the same sponge species and genetic clusters, and even more importantly, that those are acting over the same bacterioplankton community (i.e., same location) that is being filtered and acquired through horizontal strategies. However, *P. ficiformis* microbial communities were less similar across basins (Eastern and Western basins of the Mediterranean) even when the same genetic cluster appeared in both basins. The availability of the specific microbial variants in the environment could have affected the fidelity of the sponge microbiomes in such distant locations, since the planktonic communities are affected by very particular ocean circulation patterns in both basins (Elshanawany et al., 2010; Mapelli et al., 2013). In fact, a previous study about the intra‐specific variation of *P. ficiformis* found that for their violet morph (sponges presenting Chroloflexi), samples from Italy and Israel harboured highly different communities (Burgsdorf et al., 2014). This said, however, we noticed that the most Eastern location, ISR, could belong to a highly divergent population, as indicated in a more detailed hierarchical analyses of genetic cluster assignation (i.e., ISR was identified as a different genetic cluster (Pf10) instead of the shared Pf5, Figure S7). Therefore, it is unclear whether differences can be attributed to variable communities of planktonic microbes within this location or to the presence of a divergent sponge lineage in ISR.

Contrastingly, *Ircinia fasciculata* and *Dendrilla antarctica* presented high gene flow among the locations sampled, showing very subtle, host genetic substructure. While *I. fasciculata* transmits part of their microbiome vertically (Björk et al., 2019), *D. antarctica* seems to rather acquire its microbiome horizontally from the surrounding waters (Koutsouveli et al., 2018). Both these sponges have higher predicted larval dispersion rates than those of *P. ficiformis* (Maldonado & Riesgo, 2009; Mariani et al., 2005). Indeed, correlations between the microbial community dissimilarity and the host genetic distance were weak, and the variation seemed more influenced by the spatial distance. For individual locations, only one site for *D. antarctica* and two for *I. fasciculata* presented positive correlation between the host genetic distance and the microbial dissimilarity. It is possible that, due to the low genetic differentiation among the populations of these species (i.e., high gene flow) and therefore strong speciation constraints, their allele content and frequencies in host loci responsible for symbiont selection are not fixed. Moreover, site‐specific environmental conditions could amplify or mask the effects of related host genes on the microbiome composition, giving variable results in different locations, as previously noted for plant species (Wagner et al., 2016). In fact, *I. fasciculata* presented larger and more variable proportions of site‐specific ASVs than the other species, supporting the idea of a stronger but variable effect (among locations) of the surrounding water in the microbial community of this species. Interestingly, studies on the microbiome composition and stability in *Ircinia* species (Erwin, et al., 2012; Pita et al., 2013) showed that their microbiome was stable at large geographical scales and over time, although the lower sequencing depth of their approach could have not captured the variation. At latitudinal scales (2,800 km), however, *Ircinia campana* displayed different microbial communities correlated with the geographic distance. In that study, two host haplotypes were identified (using partial COI gene sequences) but these also presented a latitudinal distribution, not allowing to discriminate the effect of each factor in the microbiome dissimilarity (Marino et al., 2017).

Two recent studies have used microsatellites to identify population structure of single sponge species. *Iricinia campana* (*F*_ST_ = 0.021) was again the focus of one of them, and the authors reported that microbiomes significantly differed in composition between locations, and that within individual locations variability was correlated with host genetic distance (Griffiths et al., 2019). Unfortunately, the effect of host genetic clusters and location in *I. campana* was not assessed together in that study neither (Griffiths et al., 2019). For *Cliona delitrix* (*F*_ST_ = 0.158), populations were also confined to specific sampling locations, preventing the decomposition of genetic and geographic effects, however, an analysis restricted to the two populations present in more than one site showed lack of spatial correlation but a significant correlation with host genetic distance (Easson et al., 2020), similarly to our results for *P. ficiformis*.

### 4.2 Sponge microbiome composition: Specific and core taxa across species and genetic clusters

*Petrosia ficiformis* and *I. fasciculata* are both considered high microbial abundance (HMA) sponges (Erwin et al., 2012, 2015), while *D. antarctica* can be considered a low microbial abundance (LMA) sponge based on our results (Figure S1) and microscope observations (Koutsouveli et al., 2018). The particular composition of each sponge species was similar to previous descriptions of *P. ficiformis* (Burgsdorf et al., 2014; Schmitt et al., 2012; Sipkema et al., 2015) and *I. fasciculata* (Erwin, et al., 2012). The microbiome of *D. antarctica* has not been described previously. Interestingly, *P. ficiformis* and *I. fasciculata* shared 21 phyla, with different relative abundances, while 10 phyla out of the 22 present in *D. antarctica* (including Patescibacteria, Planctomycetes and Nitrospirae, most of them in low abundance), were not shared with the Mediterranean sponges.

In global analyses of the microbiome in Porifera, symbiont taxa found in many species (core microbiome) account for a small part of the community, while a much larger portion of the community is usually host species‐specific (Schmitt et al., 2012; Thomas et al., 2016). Here, without exception, very few ASVs were present in all samples of a species, although, using a less strict definition of 80% of samples, we recognised 55, 41, and 37 core ASVs for *P. ficiformis*, *D. antarctica* and *I. fasciculata*, respectively. The relative abundance that these core ASVs represented was usually high, up to 43% and 49.8% of the whole microbiome for *D. antarctica* and *I. fasciculata*, respectively. In *P. ficiformis*, the values were generally lower, with a maximum relative abundance of 33%, already showing that its microbiome was more variable than the other two species.

Interestingly, the dominant genera in the sponges included both core ASVs (generalists, i.e., microbes present in most of the individuals) as well as indicator ASVs (specialists, i.e., microbes specific to a genetic cluster). Core ASVs accounted for a high percentage of the community, while indicator ASVs usually comprised low abundances. These results indicate that ASVs with opposite behaviour (i.e., generalist versus specialists), are very closely‐related microbial variants, instead of distant taxa. It has been proposed previously that different sponges contain different bacterial species, however, these bacteria are still closely related to each other explaining the co‐evolution patterns observed in bacterial communities of sponges (Montalvo & Hill, 2011; Schmitt et al., 2012). This phenomenon was first described by Hentschel et al. (2002) who showed that sponge‐derived 16S rRNA gene sequences cluster together regardless of their origin (host sponge and/or sampling location). Similar to our results, oligotype analysis of closely‐related *Nitrospira* revealed that some *Nitrospira* variants were differentially enriched in closely related sponge species, and different from other distant sponges or seawater (Reveillaud et al., 2014). In summary, sponges therefore contain a uniform, sponge‐specific microbial community if we look at higher taxonomic levels, but each sponge species contains different microbial variants.

All this together highlights the caveats associated to the analysis of microbiome composition at these higher taxonomic levels (OTU at 97% or genus level), which may contribute to hide species‐specific patterns in microbial species. The genus level analysis in our *P. ficiformis* samples could not detect differences in the microbial communities of Pf3 compared to the other genetic clusters, while the ASV level analysis did. Moreover, <1% ribosomal variation can include large differences in gene content, representing strains with differing metabolic capabilities (Ansorge et al., 2018; Koehorst et al., 2018). ASV methods have demonstrated sensitivity and specificity as good or better than OTU methods and better discriminate ecological patterns (Callahan et al., 2017; Eren et al., 2015; Needham et al., 2017; Tikhonov et al., 2015), which is why they were used in this study.

### 4.3 Relative importance of horizontal versus vertical transmission of the sponge microbiome

Two of the sponge species studied here presented exclusive horizontal transmission of symbionts (larvae devoid of microbes), however their microbiome was still very specific to the hosts, suggesting that high species‐specificity is not dependent of the vertical transmission of symbionts to offspring in sponges. Recent advances to understand the role of vertical transmission on the specificity of the microbiome have shown that the vertical transmission of the microbiome from the parents to the larvae is incomplete, with larvae harbouring less than 50% of the microbes from their parents, and siblings sharing only 17% of microbes among them (Björk et al., 2019). Also, regardless of the microbial cargo of the larvae, the symbiont community will fluctuate when juvenile sponge starts pumping, and it will be stabilised later on with a different composition from that of the swimming larvae (Fieth et al., 2016; Sacristán‐Soriano et al., 2019). All this suggests that the main strategy to acquire the specific microbiome observed in adult sponges is horizontal transmission, and evidence is accumulating in this direction (Nguyen & Thomas, 2018; Turon et al., 2018).

In sponges, the way to achieve this host‐specificity could be through very specific recognition systems to discriminate symbionts taken from the water, and through a complex immune system (Hentschel et al., 2012; Pita et al., 2018; Riesgo et al., 2014). For instance, differences in the subset of pattern‐recognition receptors (PRRs) of the innate immune system may relate to differences in the microbial composition in sponges (Pita et al., 2018). Other genes could also play a role, like in plants, where host loci for defence and cell wall integrity have been identified as responsible of differences in the phyllosphere microbiome (Horton et al., 2014), but this is largely unexplored in sponges. We suggest that these molecular mechanisms, at least for the species *P. ficiformis*, might be population‐specific rather than species‐specific, given the primary role of the genetic cluster in shaping the symbiont microbial structure. Certainly, more studies are needed to test the link between microbial communities and host genetic cluster in sponges in order to establish whether this is a common pattern, but *P. ficiformis* emerges as a fundamental model to understand this link.

In conclusion, our large‐scale approach with 393 samples of three different species with contrasting dispersal potential and strategies for microbiome acquisition allowed us to discover the role of the sponge host genetics at the intraspecific level influencing the microbiome structure and composition for the first time. We observed that within species, microbial communities specific to different genetic clusters can also be identified, but the extent of the host influence compared with a spatial effect was entirely dependent on the gene flow among populations and therefore their genetic differentiation (fixation index). When gene flow is restricted, the effect of host genetic cluster is larger than when gene flow allows relatively homogenous genomic pools across locations. Our study provides fundamental insight to understand when microbiomes become specific in sponges, highlighting the necessity of taking into account the complex evolutionary history of each species.

## AUTHOR CONTRIBUTIONS

C.D.V., and A.R. conceived the study. A.R, S.T, and C.L. performed the fieldwork. C.D.V., K.B., and C.L. performed the laboratory work, and C.D.V. analysed the data. C.D.V., and A.R. drafted the first versions of the manuscript. All authors commented on and approved of later versions of the paper.

## Supporting information

Fig S1‐S7

Table S1‐S14

## Data Availability

The raw sequences were deposited at the Sequence Read Archive (SRA) of the NCBI as BioProject with accession ID PRJNA593003. Population genetic data for *Petrosia ficiformis* is available thought https://doi.org/10.1594/PANGAEA.882098 (see Riesgo et al., 2019), for *Ircinia fasciculata* through https://doi.pangaea.de/10.1594/PANGAEA.860018 (see Riesgo et al., 2016), and SNPs data for each individual sample of *Dendrilla antarctica* is deposited in the NCBI SRA database, BioProject PRJNA531366, Biosamples SAMN11350306–SAMN11350367 (see Leiva et al., 2019).
