# Supplementary material for: On the way to specificity ‐ Microbiome reflects sponge genetic cluster primarily in highly structured populations
Source: Mol Ecol. 2020 Sep 30;29(22):4412–27. doi: 10.1111/mec.15635 (PMC7756592; doi:10.1111/mec.15635)
Supplement: Supplementary file 1 — Fig S1‐S7 [file MEC-29-4412-s001.docx]

**Supplemental Information for:**

***On the way to specificity - Microbiome reflects sponge genetic cluster primarily in highly structured populations***

Cristina Díez-Vives, Sergi Taboada, Carlos Leiva, Kathrin Busch, Ute Hentschel, Ana Riesgo

**Table of Contents:**

| **Figure S1** | Page 2 |
| --- | --- |
| **Figure S2** | Page 3 |
| **Figure S3a** | Page 4 |
| **Figure S3b** | Page 5 |
| **Figure S4** | Page 6 |
| **Figure S5** | Page 7 |
| **Figures S6 and S7** | Page 8 |

**
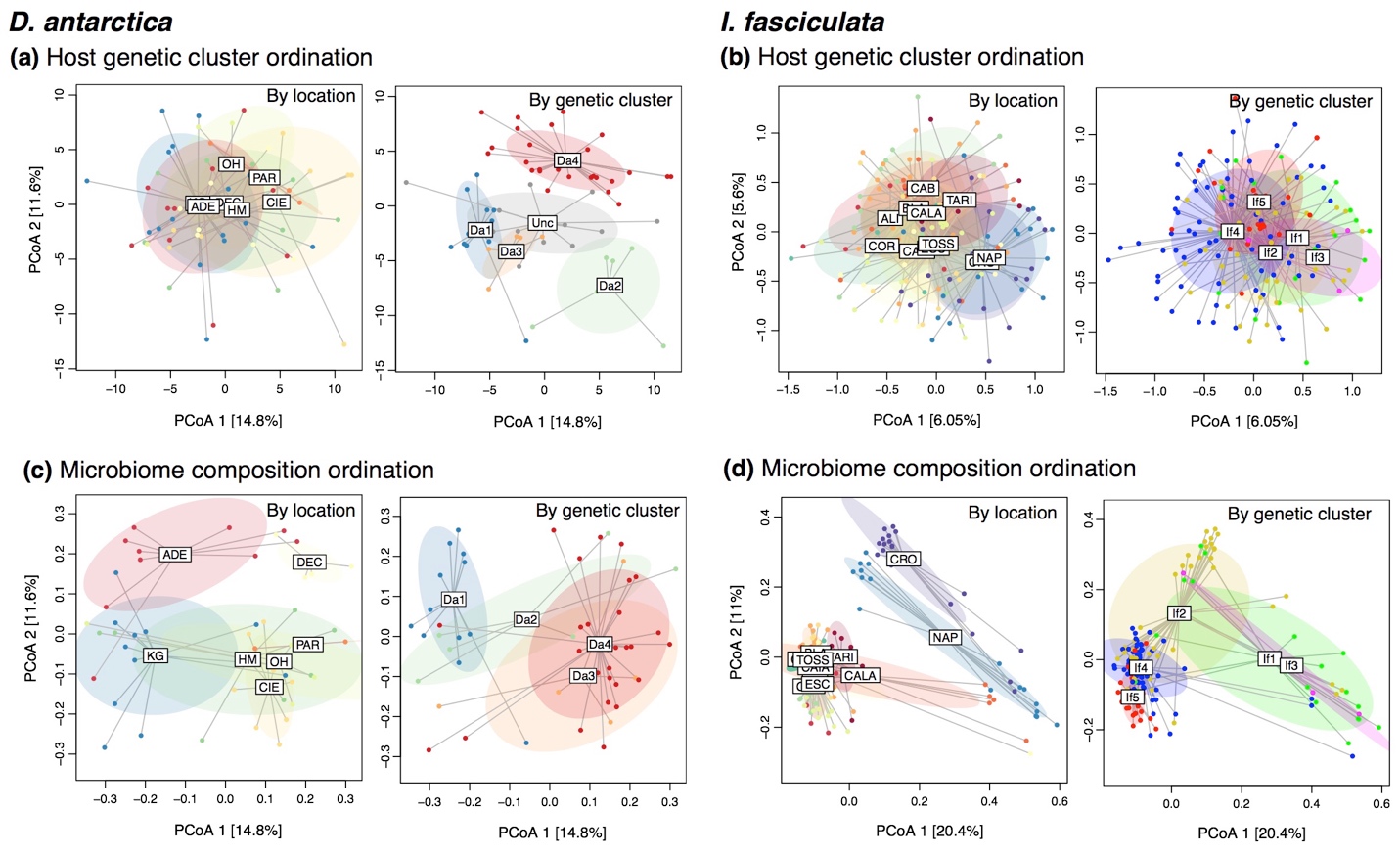
**

**Figure S1.** PCoA plots for *Dendrilla antarctica* and *Ircinia fasciculata* showing **(a-b)** ordination of the host genetic distances including all samples and colored by the corresponding location (left side) and by the assigned genetic cluster (right side), and **(c-d)** ordination of microbiome dissimilarities of all samples, and colored as before. Centroids are labelled with their respective factor label and groups are circled with 0.7 data coverage for data ellipses.

**
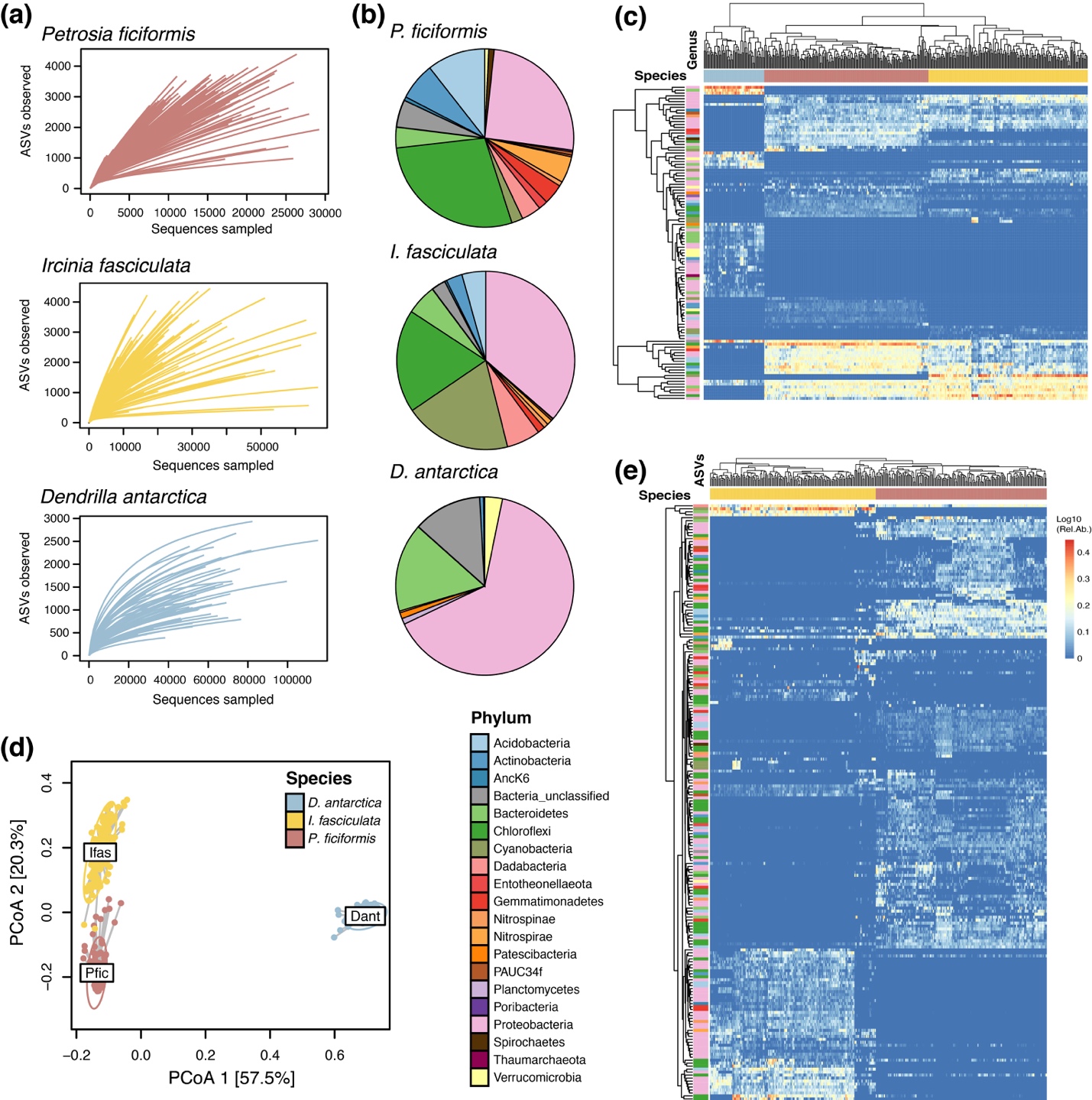
**

**Figure S2. (a)** Rarefaction curves for the three sponge species. **(b)** Pie charts showing general taxonomy (average relative abundances values across all samples) at phylum level. **(c)** Heatmaps showing log10 (relative abundances) of ASVs aggregated at genus level for the three sponge species or **(e)** directly at ASVs level for *I. fasciculata* and P*. ficiformis*. In both graphs the row information is colored by the phylum level matching the pie charts, and the columns by the species. **(d)** Ordination plot of the samples based on Bray Curtis dissimilarity of relative abundance values aggregated at genus level. Centroids are labelled with the species label and groups are circled with 0.9 data coverage for data ellipses.

**Figure S3a.** Correlation plots of microbiome dissimilarity (Bray Curtis) *vs*. host genetic distance (Euclidean) for individual locations. Locations with more than one genetic cluster with at least three replicates are shadowed in grey. The number of samples belonging to the different genetic clusters are shown within each plots, in black letters for genetic clusters with more or equal than 3 replicates, and in grey for less than 3 replicates (for *D. antarctica* the threshold is 2 replicates). Mantel test statistic (Pearson’s *r*) and *p*-values (*p*) are shown inside each plot, and significant correlations are colored in blue.

**Figure S3b.** Correlation plots of microbiome dissimilarity (Bray Curtis) *vs*. spatial distance (kilometers between locations) for individual genetic clusters. Genetic clusters present in more than one location with at least three replicates are shadowed in grey. The number of samples taking in the different locations are shown within each plots, in black letters for genetic clusters with more or equal than 3 replicates, and in grey for less than 3 replicates (for *D. antarctica* the threshold is 2 replicates). Mantel test statistic (Pearson’s *r*) and *p*-values (*p*) are shown inside each plot, and significant correlations are colored in blue.

**Figure S4.** Shannon*H* diversity values within genetic clusters of the left hand columns, and within locations on the right hand columns for the three sponge species. Statistic and significance values are given in Table S8.

**Figure S5.** Ordination plots of microbiome dissimilarity (Bray Curtis) for **(a)** individual locations and **(b)** for individual genetic clusters. Statistic and significance values can be found in Table S13.


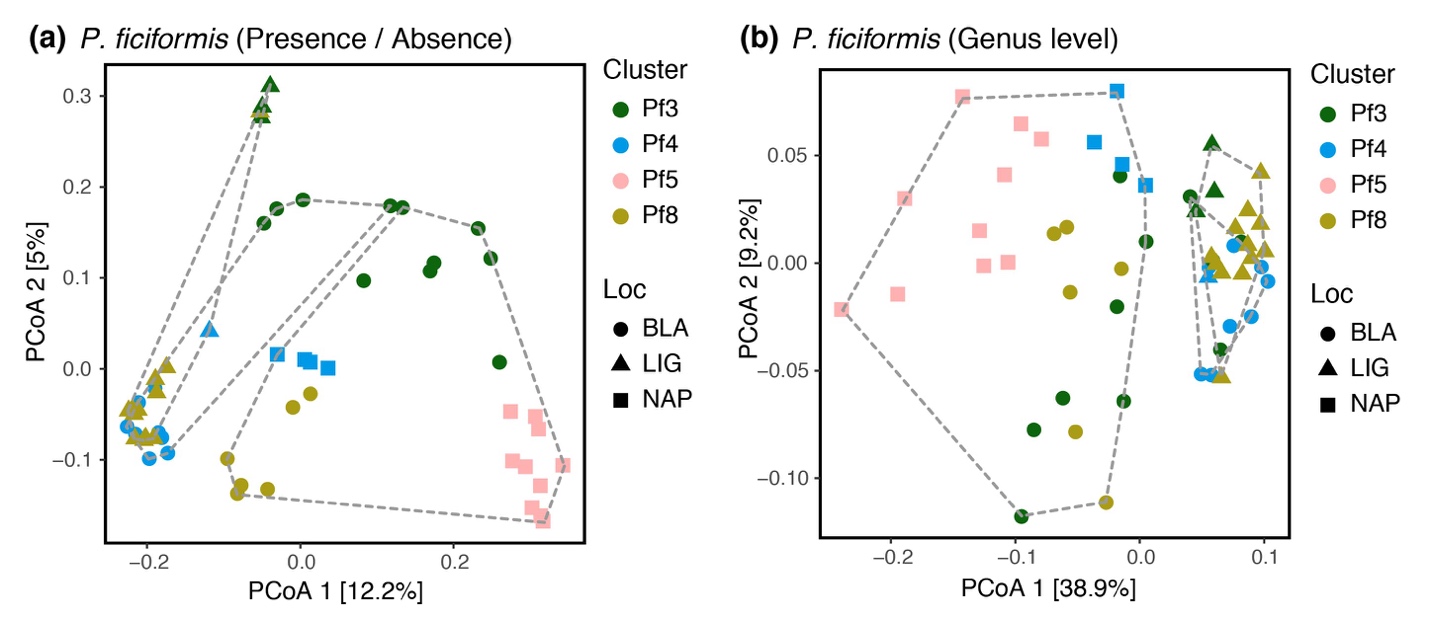


**Figure S6.** Ordination plots for the microbiome of *Petrosia ficiformis*. Bray Curtis dissimilarities were calculated on ASVs transformed to presence/absence values **(a)**, and on relative abundances aggregated at genus level **(b)**. Dotted lines circle samples from different years.


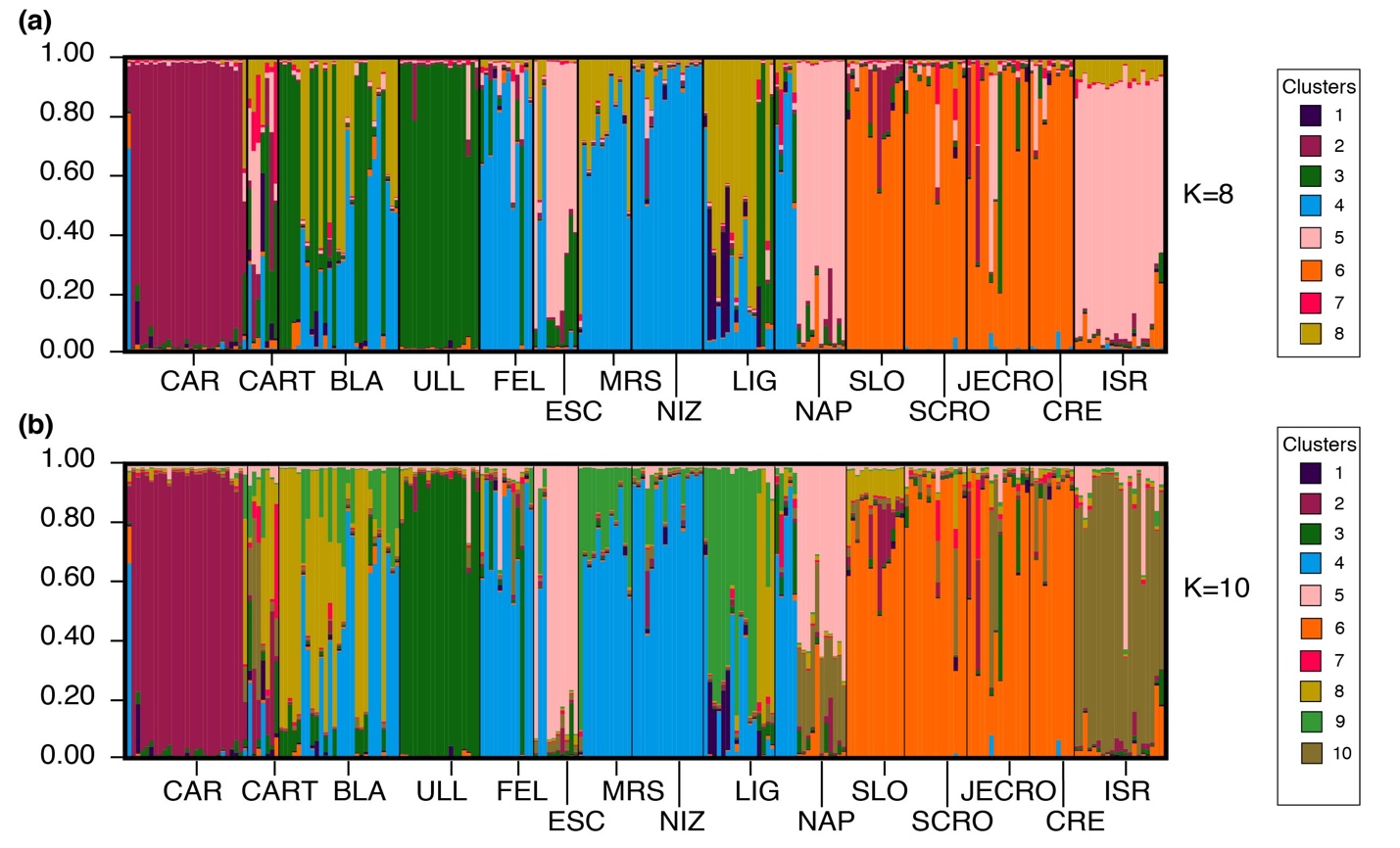


**Figure S7.** Assignment of individual genetic clusters of *P. fasciculata* to genetically similar clusters (K) as inferred by STRUCTURE for all studied locations with K = 8 **(a)** and K = 10 **(b)**.
